# Cross-evaluation of social mining for classification of depressed online personas

**DOI:** 10.1515/jib-2020-0051

**Published:** 2021-05-20

**Authors:** Alina Trifan, José Luis Oliveira

**Affiliations:** IEETA/DETI, University of Aveiro, Aveiro, Portugal

**Keywords:** cross-evaluation, depression, machine learning, post-partum depression, social monitoring

## Abstract

With the continuous increase in the use of social networks, social mining is steadily becoming a powerful component of digital phenotyping. In this paper we explore social mining for the classification of self-diagnosed depressed users of Reddit as social network. We conduct a cross evaluation study based on two public datasets in order to understand the impact of transfer learning when the data source is virtually the same. We further complement these results with an experiment of transfer learning in post-partum depression classification, using a corpus we have collected for the matter. Our findings show that transfer learning in social mining might still be at an early stage in computational research and we thoroughly discuss its implications.

## 1 Introduction

The Internet and social media have become major sources of health information, providing both broad and targeted exposure to such information, as well as facilitating information-seeking and sharing. As people increasingly turn to social media for news and information, these platforms can serve as novel sources of observational data for infodemiology, public health surveillance, as well as tracking health attitudes and behavioral intention. Because social media services collect and store a substantial amount of public information about its users, they have gained a high interest from the computational research community over the last decade. That is mainly due to the fact that they represent important sources for the extraction of health insights. The ubiquitous use of social media platforms, many of which allow users to remain anonymous, drives the opportunity for non-intrusively tracking behavioral patterns over time without disclosing personal information.

The recent pandemic, among other devastating implications, has shown that mental healthcare is one of the clinical area with most under-supply in diagnosis and care. Major challenges exist with the diagnosis, prevention and treatment of mental illnesses, and researchers are working to fill the gap and ensure universal access to quality mental healthcare services [1]. Machine learning is poised to change many fields of health, including psychology and enable more personalized healthcare provision in several areas, including mental health. The wealth of information provided by social media users in combination with machine-learning technologies have the ability to enable automated psychological assessment and diagnosis [2]. A growing body of research has begun to suggest a potential role for technology in helping to address the automated analysis and monitoring of social media language use in the mental health spectrum [3], [4], [5].

While technology cannot and should not replace the role of the clinician, it may provide clinicians personal insights into a patient’s well-being state and mood, that ultimately adds value to the care process [1]. However, the use of machine learning to diagnose people through social media is a young field with many challenges to overcome. While the large nature of data in public social mining makes it difficult for individuals to be targeted and identified, how the personal information is shared as well as what type of analysis are made have some inherent privacy concerns that only recently started being addressed. Another challenge that has only been tackled in the last couples of years is understanding to what degree these machine learning models are supported by clinical evidence and how generalized they are. In a pioneer study on the matter, Ernala et al. [6] raise awareness on these challenges that are under-explored in the current research scenario of social mining for mental health. On a more recent study, Harrigian et al. [7] conduct a study on the generalization of different machine learning approaches. Their results are not flattering for the mental health mining research community and lead to further recommendations on how we should avoid overfitting such solutions to a given dataset or problem.

In this paper we aim to contribute to understanding the challenge of approach generalization in social mining for the classification of self-diagnosed depresses users, based on their writing history on social media. As such, we conduct an exploratory study on the matter of transfer learning in mental health mining. We explore two publicly available datasets of Reddit posts of self-diagnosed depressed users with the goal of classifying depressed users. Since the two datasets contain virtually the same type of information and the source is the same, we cross-evaluate machine learning models trained on one dataset and evaluated on the other. We do so in an attempt to understand how much the machine learning models developed generalize over different datasets. Moreover, we evaluate the same models on a post-partum depression (PPD) Reddit dataset that we collected for the matter. Current scientific literature unveil little prior work focused on predicting PPD based on a mother’s social media history or usage. One possible explanation is the difficulty of prospectively engaging mothers in a screening process that accesses their digital writings, and consequently behaviors, during a physically and emotionally taxing time. Despite such challenges, we believe social media provides a rich source of information, yet to be tapped for clinical use, that reports on a person emotional well-being.

This paper is organized in five more sections. Section 2 overviews the current state of social mining in the mental health spectrum. We present the methodology we followed in Section 3. Results are presented in Section 4 and their implication is discussed in Section 5. We draw our final remarks in Section 6.

## 2 Related works

Previous research has reported that social media activity could be used to understand mental health in individuals and could also enable more personalized methods for providing timely mental healthcare. Such statements are also supported by research initiatives in the form of shared tasks, such as Conference and Labs of the Evaluation Form (CLEF) eRisk1
https://early.irlab.org/. or Computational Linguistics and Clinical Psychology (CLPsych) Workshop.2
https://clpsych.org/. These workshops appeal to an interdisciplinary audience sharing findings, models, and methods to increase access to and scalability of mental health care, by providing a closer union between clinical psychology and natural language processing. They release extensive annotated corpora, that grow each year, containing public, anonymous texts of thousands of users, retrieved from social networks and actively contribute to the development of this research area.

On the early days of social mining, several studies focusing on mental health understanding through social network data have been conducted using Twitter texts. Twitter was the preferred data source as it was the first social media network to provide an application programming interface (API) that allowed data retrieval. Coppersmith et al. [3] presented a method for gathering data for a range of mental illnesses along with proof-of-concept results that focus on the analysis of four mental disorders: post-traumatic stress disorder, depression, bipolar disorder, and seasonal affective disorder. Their ultimate goal was to enable the ethical discussion regarding the balance between the utility of such data and the privacy of mental health related information. Later on, Coppersmith et al. [4] released a Twitter dataset of users who have attempted suicide, matched by neurotypical control users. Language modeling techniques were employed to classify these users, along with open government data to identify quantifiable signals that can relate them to psychometrically validated concepts associated to suicide.

In the spectrum of social mining for the identification of depression, Harrigian et al. [7] show that the use of sentiments, emotions, and negative words in a statement is very influential in determining the level of depression. A depressed person more often uses negative words that indicate his self-despair, prolonged sadness, even suicidal thoughts. Mendu et al. [8] implemented a framework for constructing rich feature spaces from digital text communications and applied it to a dataset of private Facebook messages in a college student population. Their results show language usage in relation to validated measures of trait-level anxiety, loneliness, and personality [8]. Yadav et al. [9] explored a new dimension of social media in Twitter to identify depressive symptoms. They also introduced a robust BERT model that learns to automatically discover complementary features required to identify the symptoms with the help of the auxiliary task of figurative usage detection. Another recent model, STATENet, is a transformer based model that augments linguistic models with historical context for providing cues for suicide risk assessment on social media [10].

While there are many works on the topic of social mining for depression screening, not many of them address PPD.

De Choudhury et al. [11], [12] and more recently, Trifan et al. [13] have addressed the identification and prediction of mothers at risk of PPD using social media data coming from Facebook or Reddit. De Choudhury et al. analyzed more than just the text of a post and their prediction models took into consideration other types of interactions with the social networks, such as times and frequencies of likes and comments, among others. Their results, which were correlated to mothers’ answers to depression questionnaires, round up to accuracies up to 80%.

Following pregnancy and childbirth, PPD is a disabling but treatable mental disorder that affects approximately 10–15% of women [14]. The causes of PPD are not known. However, genetic and epigenetic susceptibility [15], [16], [17], hormonal changes [18], psychological, and social problems and especially stress [18] have been implicated. PPD is often comorbid with anxiety [19], and is strongly predicted by an antepartum history of either depression or anxiety [20], [21], [22]. Those suffering from it are at higher risks of substance abuse, anxiety, and developing chronic diseases [18], [23]. Furthermore, PPD negatively impacts the mother–infant relationship [14], [24], which can have long term consequences on the cognitive, social, and emotional development of the child [25].

Despite the widespread use of prenatal care and the increasing adoption of ante- and PPD screening tools, PPD often goes undiagnosed or untreated. For example, Ko et al. [26] estimated nearly 50% of pregnant women who experienced a major depressive episode in the past year received treatment, and Vesga-López et al. [27] estimated the prevalence for untreated, postpartum mood disorders to be 85%. Clearly, there is an opportunity to enhance existing screening tools and protocols to detect PPD, and to promote better treatments.

## 3 Implementation

In this paper we first tackle a binary classification problem of depressed online users based on two publicly available datasets. We explore machine learning algorithms along with psycholinguistic features. Since both datasets were built using posts collected from the same social network, we are interested in a cross evaluation process in order to understand the impact of the data collection process on the classification results, as well as the interoperability of such datasets. We are interested in understanding how well does a model trained on one dataset perform on a different dataset and whether this is an indicator of the generalization or reproducibility of the approach. Moreover, we explore transfer learning by evaluating one of the previous models on a PPD dataset collected from the same social network, Reddit.

### 3.1 Corpora description

Reddit is a social media network of communities that aggregate users who share a common interest for a given discussion topic. Discussion topics are called subreddits and many of them are public, meaning that anyone can read the post published within these subreddits. Reddit users are given a randomly generated username, which makes it impossible to identify a person based on the username. Moreover, it provides an API for crawling public posts. Because privacy is guaranteed and public posts can be easily collected by programmatic means, Reddit has become one of the most popular social network data source among natural language processing (NLP) researchers. For this matter, we considered Reddit a suitable and privacy insuring social media scenario for building a pilot corpus of PPD prediction based on users’ postings. The two publicly available datasets used in this work are also composed of Reddit posts and are detailed next.

The Reddit Self-reported Depression Dataset (RSDD) proposed by Yates et al. [28] consists of all Reddit users who made a post between January and October 2016. Using high-precision patterns of self-reported diagnosis, 9210 diagnosed users were matched with 107,274 control users. The Losada and Crestani test collection [29] comprises 137 depressed users matched by 755 control users. Users that expressed self-reported depression diagnoses were obtained by running specific searches against Reddit and then their writings were manually curated by the authors of the dataset. Both datasets contain the writings history of each user, from which posts that explicitly express the diagnosis were removed. We consider this curation relevant for two main reasons. First, it zeroes the possibility of wrongly classifying people engaged in social forums that share experiences of relatives or families, as well as people who might be seeking to help the ones depressed, such as doctors addressing the topic of depression. Moreover, it is relevant in the context of replicating a scenario in which depressed users do not focus on their disease and that might even be unaware of it, which turns this prediction even more ubiquitous. This relates to the possibility of identifying people that are unaware of their mental health status through heterogeneous texts.

With respect to the PPD dataset, we build a corpus of PPD related posts matched with control posts using the Reddit API.3
https://www.reddit.com/dev/api/. This API supports developers in building open source products powered by Reddit. We built a Reddit crawler using PRAW – Python Reddit API Wrapper4
https://praw.readthedocs.io/en/latest/. and scraped the following public subreddits for PPD related posts: *r/postpartumdepression*, *r/MyPPDSupport*, *r/Postpartum_Depression* and *r/PPDepression*. This process led to the collection of 512 posts, that were manually inspected and refined in three different stages. First, the 512 titles were assessed for relevance. Titles such as “Share your story here”, “Volunteers needed for a study” or containing other similar advertising information were removed. Next, for each of the PPD related post, its comments and replies were retrieved. Finally, all posts containing only a web link or short phrases such as “Thank you for the support” were removed. By the end of this third step of data cleaning we were able to harvest 491 PPD related posts. These posts were matched with 991 control ones, retrieved from a parenting subreddit – *r/beyondthebump*. The data collection process for the parenting posts ignored all posts that contained the following PPD related terms: postpartum depression, PPD and depression. We chose to retrieve parenting posts as control posts of the corpus as the research question we are posing relates to new mothers and the mother–infant relation.

### 3.2 Text preprocessing

Prior to post classification, the preprocessing of the Reddit posts followed a standard pipeline in NLP. Posts were lowercased and tokenized, all non-alphabetic characters were removed and words with less than 2 characters were filtered out, along with other stopwords. Tokenization is the process of separating a piece of text into smaller units called tokens. Here, tokens were considered words with more than two letters. Stopwords are the most common words in any natural language. For the purpose of analyzing text data and building NLP models, these stopwords do not add much value to the meaning of the text, therefore we have removed them in this pre-processing step. The NLTK stopword list was used for this purpose.5
https://gist.github.com/sebleier/554280.


### 3.3 Psycholinguistic features

The background study on the use of social media for mental health status prediction revealed a series of cognitive features that characterize the writings of depressed users. We explored the use of some of these patterns as features to be considered by a rule based estimator, which we developed with the intent of understanding their impact on the classification of depressed users. We describe next the ones on which we focused in this paper.

– Absolutist words (Table 1) – a recent study on absolutist thinking, which is considered a cognitive distortion by most cognitive therapies for anxiety and depression, showed that anxiety, depression, and suicidal ideation forums contained more absolutist words than control forums [30]. The study by Al-Mosaiwi et al. resulted in a validation of an absolutist words dictionary that was used in this paper.
– Self-related words – depressed users tend to use more often self-related words (such as: I, myself, mine) in their writings [31], [32].
– Posts length – depressed and suicidal people tend to write more words than control users [5].

**Table 1:** Absolutist words validated by Al-Mosaiwi et al. [30].

Absolutely	Constant	Every	Never
All	Constantly	Everyone	Nothing
Always	Definitely	Everything	Totally
Complete	Entire	Full	Whole
Completely	Ever	Must	

### 3.4 Binary classification

As a first approach, we followed a standard natural language processing stream for text classification. We considered Bag of Words (BoW) features and term frequency-inverse document frequency (tf-idf) feature weighting for different classifiers: multinomial naive Bayes, passive aggressive classifier and support vector machine (SVM) with Stochastic Gradient Descent. A second approach took into consideration the psycholinguistic features previously introduced, that we modelled as features of a rule-based estimator. We then considered a feature union of equal weights for tf-idf and the output of the rule-based estimator, combined with a passive aggressive classifier. The code was written in Python6
www.python.org. and we used scikit-learn [33] as machine learning framework.

## 4 Application

Because the two depression datasets were considerably different in size, we opted for using the one proposed by Yates et al. [28] as reference and cross evaluate its performance on the one introduced by Losada and Crestani [29]. For each datasets, we present the statistics of the training and test subsets in Tables 2 and 3 respectively.

**Table 2:** Statistics of the training datasets.

	[28]		[29]	
	Control	Depressed	Control	Depressed
Number of subjects	36197	3112	403	83
Avg. number of words per user	20820	69556	21318	16416
Avg. number of absolutist words	189	701	153	154
Avg. number of self-related words	579	2411	430	731

**Table 3:** Statistics of the test datasets.

	[28]		[29]	
	Control	Depressed	Control	Depressed
Number of subjects	36218	3112	352	54
Avg. number of words per user	21164	70305	21933	15370
Avg. number of self-related words	590	2435	529	637
Avg. number of absolutist words	189	709	167	145

These statistics show for each of the two datasets a sharp difference in the number of self-related words and the total number of words used by user. This is valid for both training and test collections. When it comes to absolutist words, we note that in the Losada and Crestani dataset there is no clear distinction between the number of absolutist words used by control and depressed users, both in the training and test collection. This explains the low recall value obtained in the classification when relying on the rule based estimator.

Table 4 presents the classification results for each of the datasets when the training was done on the respective training corpus and evaluation was measured on the corresponding test corpus.

**Table 4:** Classification results.

Method	Prec.	Rec.	F1	Acc
**[28]**				
Support vector machine	**0.76**	0.62	0.68	**0.95**
Multinomial Bayes	0.61	0.47	0.53	0.94
Passive aggressive	0.64	0.64	0.64	0.94
Feature union rule-based	0.68	**0.72**	**0.70**	**0.95**
[28] CNN	0.75	0.57	0.65	N/A
[28] FastText	0.37	0.70	0.49	N/A
**[29]**				
Support vector machine	**0.91**	0.20	0.33	**0.89**
Multinomial Bayes	0.52	**0.64**	**0.57**	0.87
Passive aggressive	0.70	0.38	0.50	**0.89**
Feature union rule-based	0.72	0.14	0.24	0.87
[29] baseline	0.62	0.59	0.65	N/A

For the Yates et al. dataset the feature union estimator provides the best results in terms of Recall and F1 score, while support vector machine leads to an improved precision. In the case of the Losada and Crestani dataset best results are obtained by the Multinomial Bayes predictor, while feature union results are probably negatively influenced by the use of absolutist words as a feature.

The results of the cross evaluation are presented in Table 5. For this experiment, we trained the model on the entire Yates et al. dataset and we considered the whole Losada and Crestani dataset as test corpus.

**Table 5:** Cross evaluation results.

Method	Prec.	Rec.	F1	Acc
Support vector machine	**0.46**	0.38	0.42	**0.83**
Multinomial Bayes	0.19	0.19	0.19	0.75
Passive aggressive	0.40	**0.62**	**0.49**	0.80

The results obtained in the cross evaluation are slightly worse than when training and testing on the same dataset. While these results are preliminary and probably intuitive for most readers, we consider such cross evaluations important for researchers to understand whether their work is useful outside a well-defined scenario. Moreover, these results represent evidence for supporting research into understanding how much does data collection and curation contribute for the prediction or detection biases.

Our final experiment targeted the relation between generalized depression and PPD. For this purpose, we employed one model trained on the RSDD. We show the results obtained with four different estimators, as well as the model trained on the RSDD in Table 6. These results demonstrate that it is possible to distinguish PPD related context from generalized user writings with quite a high degree of confidence. However, the use of a model trained on a depression dataset leads to less accurate results.

**Table 6:** Comparative results on classifying PPD. These results were obtained prior to the removal of depression related terms from the corpus.

Method	Prec.	Rec.	F1	Acc
Stochastic gradient descent (l1 = 0.95, loss = *hinge*)	0.90	0.88	0.89	**0.93**
Multinomial naive Bayes (alpha = 1)	**0.95**	0.81	0.87	0.92
Perceptron	0.83	0.87	0.85	0.89
Passive aggressive (loss = *sqrt_hinge*)	0.90	**0.89**	**0.9**	**0.93**
RSDD trained model	**0.95**	0.43	0.6	0.8

Table 7 summarizes the results obtained after removing depression related words from the collected corpus. As all four estimators led to similar results, we list only the ones obtained with the passive aggressive classifier as it performed slightly better among the four. These results show that there is more to be explored in the psycholinguistics and writing style of mothers suffering from PPD than just the simple use of depression related wording.

**Table 7:** Comparative results on classifying PPD after removing depression related words from the corpus.

Method	Prec.	Rec.	F1	Acc
Passive aggressive (loss = *sqrt_hinge*)	0.88	**0.53**	**0.66**	**0.81**
RSDD trained model	**0.91**	0.22	0.36	0.73

## 5 Discussion

The use of machine learning to diagnose people through social media is a young field with many challenges to overcome. Samples may be biased toward younger, technologically savvy individuals, yet research on psychological assessment of persons through social media using machine learning demonstrates promise [2]. Apart from this, interoperability of both datasets and computational models of social mining are only now being explored by the research community. This simple study showed that while transfer learning holds the a great research potential in terms of reproducibility and interoperability, more attention should be paid on developing computational models that could work outside of the box of the specific problem that they originally try to solve.

While this study is not free from limitations, we consider it relevant for understanding, on one hand, how data collection impacts the results of a specific detection model and what is the current status of dataset interoperability when it comes to social media writings. Data availability has always been an issue in the era of Big Data and while consistent efforts are being made in order to securely gather social media writings, we would like to understand if data collection itself is a source of bias or whether models trained on a given dataset maintain their performance and there is indeed a knowledge transfer when used on a different dataset.

On the other hand, with respect to transfer learning for PPD, we acknowledge that one limitation of our study in the PPD analysis is that the data collection focused on specific groups of Reddit users, which might not entirely replicate the complex reality of PPD. Nevertheless, we foresee the continuation of such studies as tools for pushing forward the development of the right types of early interventions, as means to provide more personalized and precise healthcare. Patients’ language on social media may provide a valuable source of information for clinicians outside the context of clinical encounters, and computational language analysis tools show significant promise [34].

Our experiments show that substantial loss occurs when transferring between datasets, but also between clinical context. This stands as a preliminary proof that researchers tend to overestimate the performance of computational models and neglect their interoperability aspects. As the social mining community aspires to introduce computational models into the clinical setting, it is imperative that we develop such models following standardized and generalized approaches, both in terms of data collection and models evaluation.

## 6 Conclusions

Social media mining has the potential of extending the definition of digital phenotyping by contributing with new insights on a person’s well-being based on her online writings. Several studies focus on developing models for early prediction and high accuracy classification of depressed online users, but there is little work done so far in ensuring study interoperability and reproducibility. Apart from building precise and fast models, we are interested in building models that can be reused even when data changes.

This study presented cross evaluation results when using one public dataset for training and a different one for testing. These results show that cross evaluation scores are lower than when training and testing on the same dataset. These results are complemented with an experiment of transfer learning, in which we evaluate a model trained on a depression corpus in the context of PPD. The results of both experiments show that there is a certain degree of loss that occurs when transferring from on corpus to another and from one clinical context to a slightly different one. Even though this outcome is somehow intuitive, we hope that this paper could start the discussion on the topic of reusability and encourage scientist to test their approaches “out of the box”.

## References

[1] Wang T , Bashir M . Privacy considerations when predicting mental health using social media. Proc Assoc Inf Sci Tech 2020;57:e244. 10.1002/pra2.244.

[2] Fleming MN . Considerations for the ethical implementation of psychological assessment through social media via machine learning. Ethics Behav 2020;31:181–92. 34248317 10.1080/10508422.2020.1817026 PMC8261642

[3] Coppersmith G , Dredze M , Harman C . Quantifying mental health signals in twitter. In: . Proceedings of the workshop on computational linguistics and clinical psychology: from linguistic signal to clinical reality 2014:51–60.

[4] Coppersmith G , Leary R , Whyne E , Wood T . Quantifying suicidal ideation via language usage on social media. In: . Joint statistics meetings proceedings, statistical computing section JSM; 2015.

[5] Coppersmith G , Ngo K , Leary R , Wood A . Exploratory analysis of social media prior to a suicide attempt. In: . Proceedings of the third workshop on computational lingusitics and clinical psychology 2016:106–17.

[6] Ernala SK , Birnbaum ML , Candan KA , Rizvi AF , Sterling WA , Kane JM , . Methodological gaps in predicting mental health states from social media: triangulating diagnostic signals. In: . Proceedings of the 2019 CHI conference on human factors in computing systems ACM; 2019:134.

[7] Harrigian K , Aguirre C , Dredze M . Do models of mental health based on social media data generalize?. In: . Proceedings of the 2020 conference on empirical methods in natural language processing: findings 2020:3774–88.

[8] Mendu S , Baglione A , Baee S , Wu C , Ng B , Shaked A , . A framework for understanding the relationship between social media discourse and mental health. Proc ACM Human Comp Interac 2020;4:1–23. 10.1145/3415215.

[9] Yadav S , Chauhan J , Sain JP , Thirunarayan K , Sheth A , Schumm J . Identifying depressive symptoms from tweets: figurative language enabled multitask learning framework. ., arXiv preprint arXiv:2011.06149 , 2020. 10.18653/v1/2020.coling-main.61.

[10] Sawhney R , Joshi H , Gandhi S , Shah R . A time-aware transformer based model for suicide ideation detection on social media. In: . Proceedings of the 2020 conference on empirical methods in natural language processing EMNLP; 2020:7685–97.

[11] De Choudhury M , Counts S , Horvitz EJ , Hoff A . Characterizing and predicting postpartum depression from shared facebook data. In: . Proceedings of the 17th ACM conference on computer supported cooperative work & social computing ACM; 2014:626–38.

[12] De Choudhury M , Counts S , Horvitz E . Predicting postpartum changes in emotion and behavior via social media. In: . Proceedings of the SIGCHI conference on human factors in computing systems ACM; 2013:3267–76.

[13] Trifan A , Semeraro D , Drake J , Bukowski R , Oliveira JL . Social media mining for postpartum depression prediction. Stud Health Technol Inf 2020;270:1391–2. 10.3233/SHTI200457. 32570674

[14] CDC . Prevalence of self-reported postpartum depressive symptoms–17 states, 2004-2005. Morb Mortal Wkly Rep 2008;57:361. 18401329

[15] Guintivano J , Arad M , Gould TD , Payne JL , Kaminsky ZA . Antenatal prediction of postpartum depression with blood dna methylation biomarkers. Mol Psychiatr 2014;19:560. 10.1038/mp.2013.62. PMC7039252 23689534

[16] Figueiredo FP , Parada AP , de Araujo LF , Silva WA , Del-Ben CM . The influence of genetic factors on peripartum depression: a systematic review. J Affect Disord 2015;172:265–73. 10.1016/j.jad.2014.10.016. 25451426

[17] e Couto TC , Brancaglion MYM , Alvim-Soares A , Moreira L , Garcia FD , Nicolato R , . Postpartum depression: a systematic review of the genetics involved. World J Psychiatr 2015;5:103. 10.5498/wjp.v5.i1.103. 25815259 PMC4369539

[18] O’hara MW , McCabe JE . Postpartum depression: current status and future directions. Annu Rev Clin Psychol 2013;9:379–407. 23394227 10.1146/annurev-clinpsy-050212-185612

[19] Metz TD , Rovner P , Hoffman MC , Allshouse AA , Beckwith KM , Binswanger IA . Maternal deaths from suicide and overdose in Colorado, 2004–2012. Obstet Gynecol 2016;128:1233. 10.1097/AOG.0000000000001695. 27824771 PMC5121076

[20] Di Florio A , Meltzer-Brody S . Is postpartum depression a distinct disorder?. Curr Psychiatr Rep 2015;17:76. 10.1007/s11920-015-0617-6. 26267038

[21] Fleming AS , Ruble DN , Flett GL , Shaul DL . Postpartum adjustment in first-time mothers: relations between mood, maternal attitudes, and mother-infant interactions. Dev Psychol 1988;24:71. 10.1037/0012-1649.24.1.71.

[22] Howard LM , Molyneaux E , Dennis CL , Rochat T , Stein A , Milgrom J . Non-psychotic mental disorders in the perinatal period. Lancet 2014;384:1775–88. 10.1016/s0140-6736(14)61276-9. 25455248

[23] Creanga AA , Berg CJ , Ko JY , Farr SL , Tong VT , Bruce FC , . Maternal mortality and morbidity in the United States: where are we now?. J Wom Health 2014;23:3–9. 10.1089/jwh.2013.4617. PMC3880915 24383493

[24] Brummelte S , Galea LA . Postpartum depression: etiology, treatment and consequences for maternal care. Horm Behav 2016;77:153–66. 10.1016/j.yhbeh.2015.08.008. 26319224

[25] O’Hara MW . Postpartum depression: what we know. J Clin Psychol 2009;65:1258–69. 10.1002/jclp.20644. 19827112

[26] Ko JY , Farr SL , Dietz PM , Robbins CL . Depression and treatment among U.S. Pregnant and nonpregnant women of reproductive age, 2005–2009. J Wom Health 2012;21:830–6. 10.1089/jwh.2011.3466. PMC4416220 22691031

[27] Vesga-López O , Blanco C , Keyes K , Olfson M , Grant BF , Hasin DS . Psychiatric disorders in pregnant and postpartum women in the United States. Arch Gen Psychiatr 2008;65:805–15. 10.1001/archpsyc.65.7.805. 18606953 PMC2669282

[28] Yates A , Cohan A , Goharian N . Depression and self-harm risk assessment in online forums. In: . Proceedings of the 2017 conference on empirical methods in natural language processing Association for Computational Linguistics; 2017:2968–78.

[29] Losada DE , Crestani F . A test collection for research on depression and language use. In: . International conference of the cross-language evaluation forum for european languages Springer; 2016:28–39.

[30] Al-Mosaiwi M , Johnstone T . An absolute state: elevated use of absolutist words is a marker specific to anxiety, depression, and suicidal ideation. Clin Psychol Sci 2018;6:529. 10.1177/2167702617747074. 30886766 PMC6376956

[31] Chung C , Pennebaker JW . The psychological functions of function words. Soc Commun 2007;1:343–59.

[32] Rude S , Gortner EM , Pennebaker J . Language use of depressed and depression-vulnerable college students. Cognit Emot 2004;18:1121–33. 10.1080/02699930441000030.

[33] Pedregosa F , Varoquaux G , Gramfort A , Michel V , Thirion B , Grisel O , . Scikit-learn: machine learning in Python. J Mach Learn Res 2011;12:2825–30.

[34] Kelly DL , Spaderna M , Hodzic V , Nair S , Kitchen C , Werkheiser AE , . Blinded clinical ratings of social media data are correlated with in-person clinical ratings in participants diagnosed with either depression, schizophrenia, or healthy controls. Psychiatr Res 2020;294:113496. 10.1016/j.psychres.2020.113496. 33065372

